# *Cryptosporidium proventriculi* in Captive Cockatiels (*Nymphicus hollandicus*)

**DOI:** 10.3390/pathogens12050710

**Published:** 2023-05-13

**Authors:** Mariele Fernanda da Cruz Panegossi, Giovanni Widmer, Walter Bertequini Nagata, Bruno César Miranda Oliveira, Elis Domingos Ferrari, Jancarlo Ferreira Gomes, Marcelo Vasconcelos Meireles, Alex Akira Nakamura, Thaís Rabelo do Santos-Doni, Luiz da Silveira Neto, Katia Denise Saraiva Bresciani

**Affiliations:** 1 Faculdade de Medicina Veterinária, Universidade Estadual Paulista (Unesp), Araçatuba 16050-680, São Paulo, Brazil; marielepanegossi@gmail.com (M.F.d.C.P.); m.meireles@unesp.br (M.V.M.); alex.nakamura@unesp.br (A.A.N.); 2 Department of Infectious Disease & Global Health, Cummings School of Veterinary Medicine, Tufts University, North Grafton, MA 01536, USA; giovanni.widmer@tufts.edu; 3 Escritório de Defesa Agropecuária, Coordenadoria de Defesa Agropecuária, Secretaria de Agricultura e Abastecimento do Estado de São Paulo, Lins 16400-050, São Paulo, Brazil; walter.bn@hotmail.com; 4 União das Faculdades dos Grandes Lagos (Unilago), São José do Rio Preto 15030-070, São Paulo, Brazil; bruno.9988@hotmail.com (B.C.M.O.); elisd.ferrari@yahoo.com.br (E.D.F.); 5 Faculdade de Ciências Médicas e Instituto de Computação, Universidade Estadual de Campinas (UNICAMP), Campinas 13083-887, São Paulo, Brazil; jgomes@ic.unicamp.br; 6 Instituto de Ciências Agrárias (ICA), Universidade Federal dos Vales do Jequitinhonha e Mucuri (UFVJM), Avenida Universitários, Unaí 36830-000, Minas Gerais, Brazil; rabelo.vet@hotmail.com; 7 Imunologia e Vacinologia, curso de Engenharia de Bioprocessos e Biotecnologia, Universidade Federal do Tocantins (UFT), Gurupi 77410-530, Tocantins, Brazil; luiz.silveira@uft.edu.br

**Keywords:** cryptosporidiosis, birds, epidemiology, molecular characterization, 18S rRNA gene, prevalence, risk factors

## Abstract

Cockatiels (*Nymphicus hollandicus*) are among the most commonly sold psittacines pets. The aim of this study was to evaluate the occurrence of *Cryptosporidium* spp. in domestic *N. hollandicus* and identify risk factors for this infection. We collected fecal samples from 100 domestic cockatiels in the city of Araçatuba, São Paulo, Brazil. Feces from birds of both genders and older than two months were collected. Owners were asked to complete a questionnaire to identify how they handle and care for their birds. Based on nested PCR targeting the 18S rRNA gene, the prevalence of *Cryptosporidium* spp. in the cockatiels sampled was 9.00%, 6.00% based on Malachite green staining, 5.00% based on modified Kinyoun straining, and 7.00% when the Malachite green was combined with Kinyoun. Applying multivariate logistic regression to test the association between *Cryptosporidium proventriculi* positivity and potential predictors showed that gastrointestinal alterations was a significant predictor (*p* < 0.01). Amplicons from five samples were sequenced successfully and showed 100% similarity with *C. proventriculi*. In summary, this study demonstrates the occurrence of *C. proventriculi* in captive cockatiels.

## 1. Introduction

*Cryptosporidium* parasites develop in intestinal and respiratory epithelial cells of vertebrates [1,2]. Clinical manifestations range from asymptomatic [3,4,5] to potentially fatal gastroenteritis [6]. Currently, at least 44 species and more than 120 genotypes of *Cryptosporidium* have been described [2].

Few studies have examined the occurrence of *Cryptosporidium* species in cockatiels in different countries or used molecular tools to identify the species. *Cryptosporidium avium*, *C. meleagridis*, and *C. proventriculi* are the species detected most commonly in psittaciforms [2,7,8,9]. 

Cockatiels (*Nymphicus hollandicus*) belong to the order Psitaciformes, family Cacatuidae. In Brazil, these birds are kept as pets and can be bought in aviaries and pet shops. *N. hollandicus* is originally from Australia. These birds became popular because of their beauty and docility. Close contact between Psittacines, principally cockatiels, and humans raises the possibility that *N. hollandicus* can act as a reservoir of zoonotic agents [10,11,12,13,14].

Considering the potential importance of these birds to their owners and the scarcity in the scientific literature on cryptosporidiosis in domestic cockatiels, the aim of this study was to determine the prevalence of cryptosporidiosis in domestic *N. hollandicus*, to analyze risk factors of infection, and to compare the sensitivity of microscopy with nested polymerase chain reaction (nPCR) targeting the 18S rRNA gene for the detection of this parasite.

## 2. Materials and Methods

### 2.1. Ethics Committee Approval

The study was approved by the Animal Use Ethics Committee (CEUA) of São Paulo State University (UNESP), School of Veterinary Medicine, Araçatuba, Brazil, protocol FOA 2015-00358.

### 2.2. Sample Size Calculation

The minimum sample size at the 95% confidence level and with absolute accuracy of 10% was calculated to be 96 samples. Since little is known about *Cryptosporidium* prevalence in *N. hollandicus*, a population proportion of 50% was assumed [15]. A total of 100 samples were collected to obtain the desired statistical power.

### 2.3. Study Population and Fecal Sample Collection

We collected fecal samples from 100 domestic cockatiels in the city of Araçatuba, São Paulo, Brazil. In this sample, 30 birds were male, 27 female, and 43 of unknown gender. Eleven birds were <12 months old, and 89 were adults (>12 months old). A total of 50 birds were from breeders, 21 from owners, and 29 from pet shops. Samples were collected on 3 alternating days from 57 individually caged cockatiels and from 43 pools of 2 to 4 cockatiels living in the same cage. Fecal samples were collected from the bottom of the cage using a disposable wooden spatula and stored at 4 °C in 2 mL microtubes.

### 2.4. Epidemiological Questionaire

Following a standardized questionnaire, information on each bird was obtained from the owners. The owners were asked about the age (<12 months old or >12 months old), body score (skinny or normal), vermifugation (yes or no), respiratory clinical signs (sneeze: yes or no), gastrointestinal alterations (diarrhea: yes or no), contact with other animals (yes or no), frequency of cage cleaning (every day or weekly), cleaning agent used (yes or no), environment (cage, free, or both), origin (breeders, owners, or pet shops), sex (female, male, or unknown gender), drinking water (drank tap or filtered or mineral water), disposal of fecal material, environment, and nutritional management. All the interviewees signed a free consent form authorizing the use of their birds for this study.

### 2.5. Fecal Sample Purification, Microscopy and DNA Extraction

The fecal samples were homogenized in Sheather’s solution (54 g of table sugar, 355 mL of phosphate buffered saline (PBS) pH 7.4, 0.1% Tween 20) and centrifuged at 800× *g* for 5 min. The supernatant was subjected to two washes with PBS/0.1% Tween 20 and PBS/0.01% Tween 20, respectively, resulting in two aliquots of sediment which were fixed with 10% formaldehyde for microscopy or frozen to −20 °C, for DNA extraction, respectively. For microscopic identification of oocysts, fecal smears were stained with malachite green and Kinyoun modified staining [16,17]. The aliquots designated for DNA extraction were processed using the QIAamp® DNA Stool Mini Kit (Qiagen, Germantown, MD, USA), according to the manufacturer’s guidelines.

### 2.6. Nested-PCR

The nPCR protocol amplified a ~826–840 basepair (bp) fragment of the *Cryptosporidium* 18S rRNA gene [18]. Genomic DNA of *C. serpentis* and ultrapure water were used as positive and negative controls, respectively. The amplified fragments were subjected to 1.5% agarose gel electrophoresis, stained with GelRed® (Biotium, Fremont, CA, USA), and visualized with an ultraviolet light transilluminator.

### 2.7. Amplicons Sequencing

Amplicons were purified using the QIAquick Gel Extraction kit (Qiagen) and sequenced using the ABI Prism Dye Terminator Cycling Sequencing kit (Applied Biosystems, Foster City, CA, USA) in an automated ABI 3730XL sequencer (Applied Biosystems, Foster City, CA, USA). Amplicons were sequenced in both directions using the nested primers. The consensus sequence was inferred using Codoncode Aligner version 4.0.1 (CodonCode Corporation Dedham®, MA, USA). Consensus sequences were aligned to homologous sequences downloaded from Genbank with ClustalW [19] and the BioEdit sequence alignment editor [20].

### 2.8. Statistical Analysis

Statistical analyses were performed using programs STATA/SE, Version 16.1, Software (Stata Corp LLC, College Station, TX, USA), MedCalc® Statistical Software version 19.5.3 (MedCalc® Software Ltd, Ostend, West Flanders, BE), and Minitab 16.2 (Minitab Inc., State College, PA, USA). Statistical significance level was set at ≤0.05.

For inferential statistics, the presence of *Cryptosporidium* was considered the dependent variable, and other factors were considered the explanatory or independent variables. Chi-square or Fisher’s exact test were used for evaluation of the statistical significance of association between the variables investigated in the epidemiological questionnaire. To investigate the independent risk factors of each explanatory variable, all variables that showed a *p* value of ≤0.25 in a univariate analysis were analyzed with multivariate logistic regression [21]. It is advised to use an initial screening *p* value cut-off point of 0.25, as the more traditional probability of 0.05 can fail to recognize variables known to be important. The occurrence probability ratio (odds ratio, OR) and the corresponding 95% confidence intervals (CI) were calculated using univariate and multiple logistic regression. A *p* value of <0.05 was considered as the level of statistical significance for all tests.

We compared the sensitivity and specificity of the test for diagnosing *Cryptosporidium* in captive cockatiels. We compared microscopic examinations (Malachite green, Kinyoun modified, and Malachite green + Kinyoun modified) with nPCR (Table 1). First, cluster variable analyses were conducted using Ward’s linkage method to better understand the association between the tests used in the diagnosis of *Cryptosporidium*. Statistical significance of sensitivity and specificity between tests were evaluated using the chi-square or Fisher’s exact test. The performance of each test was evaluated according to the sensitivity (Se), specificity (Sp), positive predictive value (PPV), negative predictive value (NPV), disease prevalence (Pr), area under the ROC curve (AUC), and accuracy (AC). We also performed other statistical tests, namely positive likelihood ratios (PLR), negative likelihood ratios (NLR), and Cohen’s kappa coefficient (κ). CI at the 95% level were estimated for all statistical tests. Likelihood ratios (LRs) constitute one of the best ways to measure and express diagnostic accuracy. LR quantifies the increase in knowledge of the presence of a condition (infection) through the application of a diagnostic test. By agreement, marked changes in pre-test probability can be assumed for PLR exceeding 10.0 and for NLR lower than 0.1, as 2.0 and 0.5 comprise the minimally useful suggested values for PLR and NLR, respectively [22]. Area under the receiver operating characteristic (ROC) curve (AUC) was calculated in these models to determine if there was a statistically significant difference in AUCs between the diagnostics tests. The accuracy was determined in three categories: high (0.9 < AUC ≤ 1), moderate (0.7 < AUC ≤ 0.9), and, finally, low (0.5 < AUC ≤ 0.7) [23].

Cohen’s kappa coefficient (κ) was interpreted according to the following scale; κ values between 0.01 and 0.20 indicate slight agreement, 0.21 to 0.40 fair, 0.41 to 0.60 moderate, 0.61 to 0.80 substantial, and 0.81 to 1 almost perfect agreement [24].

## 3. Results

The analysis of 100 captive cockatiel samples revealed a positivity for *Cryptosporidium proventriculi* of 9.00% (n = 9). Microscopic examination and nPCR results are summarized according to sex, age, and origin in Table 1. This table also includes Pearson correlation results. The positivity of *C. proventriculi* was higher in females, adults (>12 months old), and breeders. For all evaluated samples, disposal of fecal material was indicated as regular trash. Therefore, this explanatory variable was not further analyzed.

The answers to the questionnaire provided by owners were considered categorical variables. In Table 2, the results are summarized for univariate analyses of explanatory variables for positivity of *C. proventriculi* by nPCR. This analysis identified gastrointestinal alterations as significantly associated with infection (OR 7.1, *p* = 0.017).

The multivariate logistic regression model (Table 3) of the predictors of *C. proventriculi* infection in captive cockatiels was performed with all variables that showed a *p* value of ≤0.25 in univariate analysis. Applying multivariate logistic regression analysis, a test of association between positivity of *C. proventriculi* and potential predictor showed that gastrointestinal alterations (adjusted OR 23.05, *p* = 0.003) was a significant predictor.

Table 4 lists performance of the Malachite green, Kinyoun modified, and Malachite green + Kinyoun modified when compared to nPCR as the reference standard for *C. proventriculi*. The microscopic examinations showed higher sensitivity (>98%), but lower specificity (55.6–66.7%). The accuracy was 95.0 and 96.0%.

**Figure 1:**
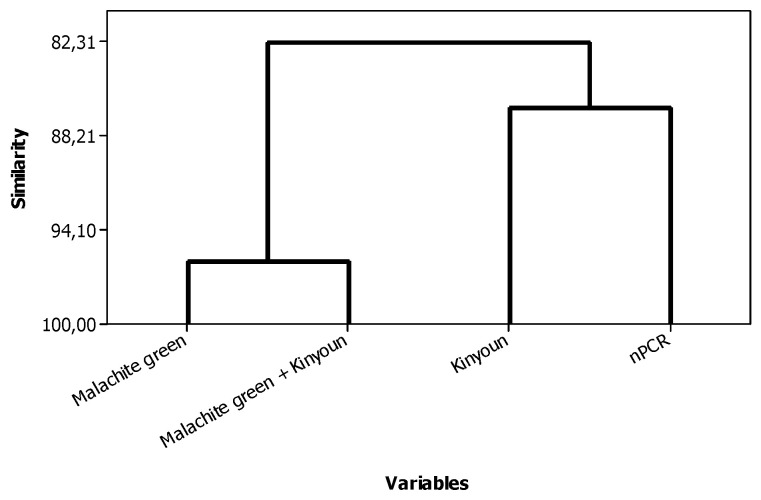
Cluster analysis of microscopy (Malachite green; Malachite green + Kinyoun modified; Kinyoun modified staining) and nPCR for detecting *C. proventriculi* in feces of cockatiels.

**Figure 2:**
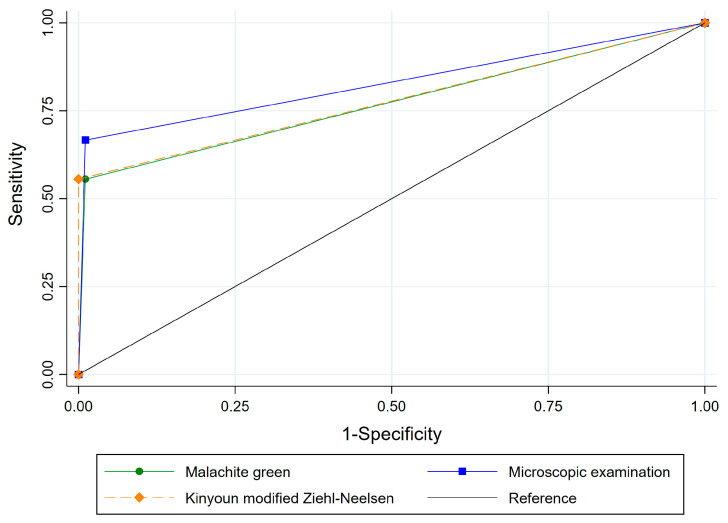
ROC curve analysis of microscopy (Malachite green; Malachite green + Kinyoun modified; Kinyoun modified staining) and nPCR for detecting *C. proventriculi* in feces of cockatiels.

Figure 1 shows the results of the cluster analysis between all diagnostics analyzed, and it can visualize the most similarity of the Kinyoun and nPCR. The association of microscopic examinations (Malachite green + Kinyoun modified) showed PPV (85.7%), NPV (96.8%), PLR (60.7), and low NLR (0.3). AUC was 0.83 (*p* < 0.01), which showed a moderate accuracy (96%) (Figure 2). All these showed kappa index (0.64 to 0.73) and were classified as agreement substantial. 

## 4. Discussion

The overall occurrence of *Cryptosporidium* found in this study is similar to those reported in the Czech Republic and Slovakia (13.7%) [4], Brazil (6.3%) [7], and China (4.5–20.5%) [5,25]. Five sequenced amplicons were 100% similar to *C. proventriculi*, the main species found in cockatiels [4,5,7,8,26,27,28,29]. *C. proventriculi* infection in this host was first reported in Australia [8], and new cases were described in Japan [26], Brazil [7,27,29], China [5,28], Czech Republic, and Slovakia [4]. Occasionally, cockatiels can be infected with *C. meleagridis* in Japan [3,26], *C. baileyi* in Japan [3], Czech Republic, and Slovakia [4], *C. avium* in Japan [26,30] and China [5,25], *C. galli* in Brazil [7,29,31], *C. ornithophilus* in Australia [8], and *C. parvum* in Brazil [7]. 

We defined “true positive” for samples that showed positive results by at least one of two different staining and nPCR techniques. There were six cases diagnosed positive by at least one technique. The low sensitivity of the microscopy observed in our study could be due to the low level of oocysts in the samples. The microscopic examinations failed to detect two true positive cases, which were probably derived from samples with low concentration of oocysts and the variable staining characteristic of this parasite and the low-grade infection of some of the birds. In spite of the lower sensitivity of Kinyoun modified, this technique was considered the gold standard by some of the earlier workers due to the direct demonstration of the organism and unambiguous diagnosis [32].

The high specificities found in this research can be explained by the experience of the observer. Observed experience, skill, and knowledge are fundamental in the screening of samples, especially in cases of microscopic examinations, as it can increase the sensitivity, specificity, and reduce to some extent the subjective error, which may be the probable cause of variation in results between different studies [33].

We found a significant association between gastrointestinal disorders and occurrence of *C. proventriculi* oocysts in feces (Table 2), but the clinical relevance of this protozoan in *N. hollandicus* remains controversial. The symptoms in this host range from asymptomatic [3,4,5] to potentially fatal gastroenteritis [6]. An outbreak of cryptosporidiosis in these birds was reported in an aviary from Korea. At the time, the aviary had 500 couples of cockatiels. Almost all chicks died within one month after hatching. The clinical signs were severe diarrhea, dehydration, depression, and ruffled feathers. Six chicks were euthanized, and the histopathological exam revealed purulent enteritis, pale and thin intestinal mucosa, villous atrophy, detachment of enterocytes, and the presence of cryptosporidial organisms in villi. *Cryptosporidium* infection was confirmed by immunofluorescence staining [6]. In Japan, one cockatiel infected with *C. avium* presented with loss of appetite, bloody diarrhea, severe emaciation, and paleness, but the clinical signs were probably caused by intestinal mucosa lesions induced by *Ascaridia nymphii*, a new nematode described by the authors [30]. In another case reported from the United States, six cockatiels developed diarrhea, four died and one was euthanized for microscopical analysis. The histopathological exam revealed cryptosporidial organisms in the microvillus border of intestinal enterocytes, but no inflammatory infiltrate was observed [34]. One case of non-purulent enteritis has also been reported, but the cockatiel died of pneumonia caused by inhalation of food [35].

A low number of *Cryptosporidium* oocysts were found (two to five per slide) during the three days of sampling. Microscopy using negative staining with malachite green presented lower positivity than nPCR. The same has been reported in other surveys on Passeriformes [36] and Psittaciformes [37], probably due to the fact that for the execution of this technique, a small amount of sample is used (around 20% of the total purified sample), potentially reducing sensitivity [16].

The lack of proper hygiene when cleaning cages may contribute to spreading the infection among captive cockatiels. Brushes used to clean cage trays were also used for cleaning drinking fountains and feeders. The use of fomites, such as bushings, to remove residues from cages can facilitate transmission of *Cryptosporidium* oocysts. Since the oocysts are extremely resistant to the action of chlorine [38], cleaning with common cleaning products may not be effective [39]. Aged birds can defecate in their feeder and drinking fountain, favoring reinfection. The lack of proper hygiene of the utensils used by the infected bird contributes to the fact that it continues to ingest their own fecal oocysts. The bushes used to clean cage trays were also used for cleaning drinking fountains and feeders, which may represent a risk of transmission of infectious oocysts.

The source of water was not a risk factor of *C. proventriculi* infection in cockatiels (*p* > 0.05). Although all positive cockatiels, except one, had ingested vegetables or tap water, the statistical analysis demonstrated that these two variables were not risk factors for *Cryptosporidium* infection (*p* > 0.05), but it is important to emphasize that the sample size included in this study is not sufficient for more reliable conclusions.

Water is an important route of transmission for several biological agents, including *Cryptosporidium* spp. [40]. Oocysts of this protozoan were detected by molecular and immunological methods in domestic tap water in the Central-West Region of Brazil [41]. Among those cockatiels surveyed in this study eating greens, fruits, and vegetables, the most frequently eaten vegetables were lettuce, tomatoes, and carrots. Vegetables may eventually be irrigated with water contaminated with feces containing oocysts of the parasite or originated from places with poor basic sanitation [42]. *C. parvum* has already been described in these foods [43]. Vegetables offered to any pet, must be properly sanitized and of suitable origin.

We conclude that there is no evidence that cockatiels play a relevant role in zoonotic transmission of cryptosporidiosis, because *C. proventriculi* has not been reported in humans. There are few epidemiological surveys on the occurrence of *Cryptosporidium* in domiciled *N. hollandicus* [4,7]. Most surveys investigate cockatiels bred in commercial establishments, aviaries, parks and zoos, or in free-ranging condition. In contrast, *Cryptosporidium* infection in cockatiels has been found in all published surveys [5,7,8,25,27,28]. Therefore, it is important to investigate the occurrence and characterize molecularly this pathogen in *N. hollandicus*, because cockatiels are commonly bred as pets and their owners have contact with the feces of these birds when cleaning the cages.

## Figures and Tables

**Table 1:** Presence of *C. proventriculi* among captive cockatiels by nPCR and microscopic examination according to the sex, age, and origin.

Variables	nPCR (Gold Standard)	Microscopic Examination		
		Malachite Green	Kinyoun Modified	Malachite Green + Kinyoun Modified
Captive cockatiels (Total)	9.00% (9)	6.00% (6)	5.00% (5)	7.00% (7)
Sex				
Female	14.81% (4)	33.33% (2)	40.00% (2)	28.57% (2)
Male	3.33% (1)	33.33% (2)	0	28.57% (2)
Unknown gender	9.30% (4)	33.33% (2)	60.00% (3)	42.86% (3)
Age				
Young (<12 months old)	11.11% (1)	0	20.0% (1)	14.29% (1)
Adult (>12 months old)	88.89% (8)	100% (6)	80.00% (4)	85.71% (6)
Origin				
Breeders	55.56% (5)	50.00% (3)	60.00% (3)	42.86% (3)
Owners	33.33% (3)	33.33% (2)	40.00% (2)	42.86% (3)
Pet shops	11.11% (1)	16.67% (1)	0	14.29% (1)
Pearson correlation (Significance Level *p* < 0.0001)				
nPCR	NC ^1^	0.656	0.729	0.735
Malachite green	0.656	NC	0.715	0.921
Kinyoun modified	0.729	0.715	NC	0.836
Malachite green + Kinyoun modified	0.735	0.921	0.836	NC

^1^ NC: not calculated.

**Table 2:** Univariable analysis of risk factors associated with presence of *C. proventriculi* among captive cockatiels.

Risk Factors	PCR (*Cryptosporidium proventriculi*)		Total	OR (CI95%) ^1^	*p* ^2^
	Negative n/%	Positive n/%			
Age					
Young (<12 months old)	10 (90.91%)	1 (9.09%)	11 (11.00%)	1	0.991
Adult (>12 months old)	81 (91.01%)	8 (8.99%)	89 (89.00%)	1.126 (0.114 < OR < 8.959)	
Body score					
Skinny	8 (88.89%)	1 (11.11%)	9 (9.00%)	1	0.817
Normal	83 (91.21%)	8 (8.79%)	91 (91.00%)	1.296 (0.143 < OR < 11.726)	
Vermifugation					
Yes	16 (88.89%)	2 (11.11%)	18 (18.00%)	1.339 (0.254 < OR < 7.055)	0.730
No	75 (91.46%)	7 (8.54%)	82 (82.00%)	1	
Respiratory clinical signs					
No	10 (100%)	0 (0%)	10 (10.00%)	1	0.593
Yes (sneeze)	81 (90.00%)	9 (10.00%)	90 (90.00%)	1.111 (1.037 < OR < 1.190)	
Gastrointestinal alterations					
Yes (Diarrhea)	6 (66.67%)	3 (33.33%)	9 (9.00%)	7.083 (1.410 < OR < 35.591)	0.017 *
No	85 (93.41%)	6 (6.59%)	91 (91.00%)	1	
Contact with other animals					
Yes	45 (90.00%)	5 (10.00%)	50 (50.00%)	1.278 (0.322 < OR < 5.066)	0.727
No	46 (92.00%)	4 (8.00%)	50 (50.00%)	1	
Frequency of cage cleaning					
Everyday	56 (91.80%)	5 (8.20%)	61 (61.00%)	1	0.726
Weekly	35 (89.74%)	4 (10.26%)	39 (39.00%)	1.280 (0.322 < OR < 5.093)	
Cleaning agent used					
Yes	21 (91.30%)	2 (8.70%)	23 (23.00%)	1	0.954
No	70 (90.91%)	7 (9.09%)	77 (77.00%)	1.050 (0.203 < OR < 5.442)	
Environment					
Cage	84 (91.30%)	8 (8.70%)	92 (92.00%)	NC ^3^	0.543
Free	5 (83.33%)	1 (16.67%)	6 (6.00%)		
Both	2 (2.20%)	0 (0%)	2 (2.00%)		
Cage	84 (91.30%)	8 (8.70%)	92 (92.00%)	1	0.448
Free	5 (83.33%)	1 (16.67%)	6 (6.00%)	2.100 (0.218 < OR < 20.250)	
Cage	84 (91.30%)	8 (8.70%)	92 (92.00%)	NC	1,000
Both	2 (2.20%)	0 (0%)	2 (2.00%)		
Free	5 (83.33%)	1 (16.67%)	6 (6.00%)	NC	1,000
Both	2 (2.20%)	0 (0%)	2 (2.00%)		
Origin					
breeders	45 (90.00%)	5 (10.00%)	50 (50.00%)	NC	0.377
owners	18 (85.71%)	3 (14.29%)	21 (21.00%)		
pet shops	28 (96.55%)	1 (3.45%)	29 (29.00%)		
breeders	45 (90.00%)	5 (10.00%)	50 (50.00%)	1	0.686
owners	18 (85.71%)	3 (14.29%)	21 (21.00%)	1.500 (0.341 < OR < 6.943)	
breeders	45 (90.00%)	5 (10.00%)	50 (50.00%)	3.111 (0.345 < OR < 28.030)	0.406
pet shops	28 (96.55%)	1 (3.45%)	29 (29.00%)	1	
owners	18 (85.71%)	3 (14.29%)	21 (21.00%)	4.667 (0.450 < OR < 48.416)	0.163 *
pet shops	28 (96.55%)	1 (3.45%)	29 (29.00%)	1	
Sex					
female	23 (85.19%)	4 (14.81%)	27 (27.00%)	NC	0.335
male	29 (96.67%)	1 (3.33%)	30 (30.00%)		
unknown gender	39 (90.70%)	4 (9.30%)	43 (43.00%)		
female	23 (85.19%)	4 (14.81%)	27 (27.00%)	5.044 (0.527 < OR < 48.267)	0.179 *
male	29 (96.67%)	1 (3.33%)	30 (30.00%)	1	
female	23 (85.19%)	4 (14.81%)	27 (27.00%)	1.696 (0.387 < OR < 7.439)	0.702
unknown gender	39 (90.70%)	4 (9.30%)	43 (43.00%)	1	
male	29 (96.67%)	1 (3.33%)	30 (30.00%)	1	0.643
unknown gender	39 (90.70%)	4 (9.30%)	43 (43.00%)	2.974 (0.316 < OR < 28.035)	
Drinking water					
Drank tap	75 (89.29%)	9 (10.71%)	84 (84.00%)	NC	0.170 *
Filtered or mineral	16 (100%)	0 (0%)	16 (16.00%)		

^1^ OR: odds ratio. Reference group marked as OR = 1; CI: confidence interval. ^2^ Pearson’s chi-square. ^3^ NC: not calculated. * Significant association (*p* < 0.25).

**Table 3:** Multivariable logistic regression model of the predictors of *C. proventriculi* among captive cockatiels.

Variables	Adjusted OR ^1^	CI95%	SE ^2^	*p*-Values
Gastrointestinal alterations	23.05	2.88 < OR < 184.13	24.44	0.003 *
Origin	1.86	0.62 < OR < 5.59	1.04	0.270
Sex	1.89	0.70 < OR < 5.09	0.96	0.205
Drinking water	3.22	0.00 < OR < 1.00	5.20	0.991

^1^ OR: odds ratio. ^2^ Standard error. * Significant predictor (*p* < 0.05).

**Table 4:** Measures of diagnostic performance in captive cockatiels for diagnosing *Cryptosporidium proventriculi*.

Parameters	Microscopic Examination				Malachite Green + Kinyoun Modified (95% CI)	
	Malachite Green (95% CI)		Kinyoun Modified (95% CI)			
Se	55.6%	(23.1–88.0)	55.6%	(26.7–81.1)	66.7%	(35.4–87.9)
Sp	98.9%	(96.8–100)	100%	(95.6–100)	98.9%	(94.0–99.8)
AUC	0.772	(0.6–1.0)	0.778	(0.6–1.0)	0.828	(0.7–1.0)
PLR	50.6	(6.6–386.8)	NC ^1^		60.7	(8.2–449.69)
NLR	0.5	(0.2–0.9)	0.4	(0.2–0.9)	0.3	(0.1–0.9)
PPV	83.3%	(53.5–100)	100%	(100)	85.7%	(59.8–100)
NPV	95.7%	(91.7–99.8)	95.8%	(91.8–99.8)	96.8%	(92.2–99.6)
AC	95.0%	(90.7–99.3)	96.0%	(92.2–99.8)	96.0%	(92.2–99.8)
κ ^2^	0.64	(0.3–1.0)	0.69	(0.4–1.0)	0.73	(0.5–1.0)
Agreement ^3^	substantial		substantial		substantial	

Abbreviations: sensitivity (Se), specificity (Sp), area under the ROC curve (AUC), positive likelihood ratios (PLR), negative likelihood ratios (NLR), positive predictive value (PPV), negative predictive value (NPV), accuracy (AC), Cohen’s kappa coefficient (κ), and confidence interval (CI). ^1^ NC: Not calculated. ^2^ Cohen’s kappa coefficient. ^3^ Nested-PCR assay was the gold standard test for calculating the kappa index.

## Data Availability

The data presented in this study are available within the article.

## References

[1] Alali F., Abbas I., Jawad M., Hijjawi N. (2021). *Cryptosporidium* infection in humans and animals from Iraq: A review. Acta Trop..

[2] Ryan U.M., Feng Y., Fayer R., Xiao L. (2021). Taxonomy and molecular epidemiology of *Cryptosporidium* and *Giardia*—A 50 Year Perspective (1971–2021). Int. J. Parasitol..

[3] Abe N., Iseki M. (2004). Identification of *Cryptosporidium* isolates from cockatiels by direct sequencing of the PCR-amplified small subunit ribosomal RNA gene. Parasitol. Res..

[4] Holubová N., Zikmundová V., Limpouchová Z., Sak B., Konečný R., Hlásková L., Rajský D., Kopacz Z., McEvoy J., Kváč M. (2019). *Cryptosporidium proventriculi* sp. n. (Apicomplexa: Cryptosporidiidae) in psittaciformes birds. Eur. J. Protistol..

[5] Qi M., Wang R., Ning C., Li X., Zhang L., Jian F., Sun Y., Xiao L. (2011). *Cryptosporidium* spp. in pet birds: Genetic diversity and potential Public Health significance. Exp. Parasitol..

[6] Kwon Y., Wee S., Kook J., Lee C. (2005). Outbreak of enteric cryptosporidiosis in cockatiels (*Nymphicus hollandicus*). Vet. Rec..

[7] Nakamura A.A., Simões D.C., Antunes R.G., da Silva D.C., Meireles M.V. (2009). Molecular characterization of *Cryptosporidium* spp. from fecal samples of birds kept in captivity in Brazil. Vet. Parasitol..

[8] Ng J., Pavlasek I., Ryan U. (2006). Identification of novel *Cryptosporidium* genotypes from avian hosts. Appl. Environ. Microbiol..

[9] Sevá Ada P., Funada M.R., Richtzenhain L., Guimarães M.B., Souza Sde O., Allegretti L., Sinhorini J.A., Duarte V.V., Soares R.M. (2011). Genotyping of *Cryptosporidium* spp. from free-living wild birds from Brazil. Vet. Parasitol..

[10] Alcaraz L.D., Hernández A.M., Peimbert M. (2016). Exploring the cockatiel (*Nymphicus hollandicus*) fecal microbiome, bacterial inhabitants of a worldwide pet. PeerJ.

[11] Kim S.-H., Kwon Y.-K., Park C.-K., Kim H.-R. (2021). Identification of *Campylobacter jejuni* and *Chlamydia psittaci* from cockatiel (*Nymphicus hollandicus*) using metagenomics. BMC Genomics.

[12] Lamb S.K., Reavill D., Wolking R., Dahlhausen B. (2020). Retrospective review of mycobacterial conjunctivitis in cockatiels (*Nymphicus hollandicus*). J. Avian Med. Surg..

[13] Nga V.T., Ngoc T.U., Minh L.B., Ngoc V.T.N., Pham V.-H., Nghia L.L., Son N.L.H., Van Pham T.H., Bac N.D., Tien T.V. (2019). Zoonotic diseases from birds to humans in Vietnam: Possible diseases and their associated risk factors. Eur. J. Clin. Microbiol. Infect. Dis..

[14] Pontes P.S., Coutinho S.D.A., Iovine R.O., Cunha M.P.V., Knöbl T., Carvalho V.M. (2018). Survey on pathogenic *Escherichia coli* and *Salmonella* spp. in captive cockatiels (*Nymphicus hollandicus*). Brazilian J. Microbiol..

[15] Hajian-Tilaki K. (2011). Sample size estimation in epidemiologic studies. Casp. J. Intern. Med..

[16] Elliot A., Morgan U.M., Thompson R.C.A. (1999). Improved staining method for detecting *Cryptosporidium* oocysts in stools using malachite green. J. Gen. Appl. Microbiol..

[17] Ma P., Soave R. (1983). Three-step stool examination for cryptosporidiosis in 10 homosexual men with protracted watery diarrhea. J. Infect. Dis..

[18] Xiao L., Escalante L., Yang C., Sulaiman I., Escalante A.A., Montali R.J., Fayer R., Lal A.A. (1999). Phylogenetic analysis of *Cryptosporidium* parasites based on the small-subunit rRNA gene locus. Appl. Environ. Microbiol..

[19] Thompson J. (1997). The CLUSTAL_X Windows interface: Flexible strategies for multiple sequence alignment aided by quality analysis tools. Nucleic Acids Res..

[20] Hall T.A. (1999). BioEdit: A user-friendly biological sequence alignment editor and analysis program for Windows 95/98/NT. Nucleic Acids Symp. Ser..

[21] Bendel R.B., Afifi A.A. (1977). Comparison of stopping rules in forward “stepwise” regression. J. Am. Stat. Assoc..

[22] Jaeschke R. (1994). Users’ guides to the medical literature. JAMA.

[23] Swets J.A. (1988). Measuring the accuracy of diagnostic systems. Science.

[24] Landis J.R., Koch G.G. (1977). The measurement of observer agreement for categorical data. Biometrics.

[25] Zhang X.-X., Zhang N.-Z., Zhao G.-H., Zhao Q., Zhu X.-Q. (2015). Prevalence and genotyping of *Cryptosporidium* infection in pet parrots in North China. Biomed Res. Int..

[26] Abe N., Makino I. (2010). Multilocus genotypic analysis of *Cryptosporidium* isolates from cockatiels, Japan. Parasitol. Res..

[27] Gomes R.S., Huber F., da Silva S., do Bomfim T.C.B. (2012). *Cryptosporidium* spp. parasitize exotic birds that are commercialized in markets, commercial aviaries, and pet shops. Parasitol. Res..

[28] Li J., Qi M., Chang Y., Wang R., Li T., Dong H., Zhang L. (2015). Molecular characterization of *Cryptosporidium* spp., *Giardia duodenalis*, and *Enterocytozoon bieneusi* in captive wildlife at Zhengzhou Zoo, China. J. Eukaryot. Microbiol..

[29] Nakamura A.A., Homem C.G., da Silva A.M.J., Meireles M.V. (2014). Diagnosis of gastric cryptosporidiosis in birds using a duplex real-time PCR assay. Vet. Parasitol..

[30] Abe N., Matsuo K., Makino I. (2015). *Ascaridia nymphii* n. sp. (Nematoda: Ascaridida) from the alimentary tract of a severely emaciated dead cockatiel *Nymphicus hollandicus*. Parasitol. Res..

[31] Antunes R.G., Simões D.C., Nakamura A.A., Meireles M.V. (2008). Natural infection with *Cryptosporidium galli* in canaries (*Serinus canaria*), in a cockatiel (*Nymphicus hollandicus*), and in lesser seed-finches (*Oryzoborus angolensis*) from Brazil. Avian Dis..

[32] Ghoshal U., Ranjan P., Dey A., Ghoshal U.C. (2018). Intestinal cryptosporidiosis in renal transplant recipients: Prevalence, species detection and comparative evaluation of SSU rRNA and *Cryptosporidium* oocyst wall protein genes. Indian J. Med. Microbiol..

[33] Jaiswal V., Brar A.P.S., Sandhu B.S., Singla L.D., Narang D., Leishangthem G.D., Kaur P. (2022). Comparative evaluation of various diagnostic techniques for detection of *Cryptosporidium* infection from the faecal samples of diarrhoeic bovine calves. Iran. J. Vet. Res..

[34] Lindsay D.S., Blagburn B.L., Hoerr F.J. (1990). Small intestinal cryptosporidiosis in cockatiels associated with *Cryptosporidium baileyi*-like oocysts. Avian Dis..

[35] Goodwin M.A., Krabill V.A. (1989). Diarrhea associated with small-intestinal cryptosporidiosis in a budgerigar and in a cockatiel. Avian Dis..

[36] Camargo V.D.S., Santana B.N., Ferrari E.D., Nakamura A.A., Nagata W.B., Nardi A.R.M., Meireles M.V. (2018). Detection and molecular characterization of *Cryptosporidium* spp. in captive canaries (*Serinus canaria*) using different diagnostic methods. Rev. Bras. Parasitol. Veterinária.

[37] Ferrari E.D., Nakamura A.A., Nardi A.R.M., Santana B.N., da Silva Camargo V., Nagata W.B., Bresciani K.D.S., Meireles M.V. (2018). *Cryptosporidium* spp. in caged exotic psittacines from Brazil: Evaluation of diagnostic methods and molecular characterization. Exp. Parasitol..

[38] Xiao L., Fayer R., Ryan U.M., Upton S.J. (2004). *Cryptosporidium* taxonomy: Recent advances and implications for Public Health. Clin. Microbiol. Rev..

[39] Luka G., Samiei E., Tasnim N., Dalili A., Najjaran H., Hoorfar M. (2022). Comprehensive review of conventional and state-of-the-art detection methods of *Cryptosporidium*. J. Hazard. Mater..

[40] King B., Fanok S., Phillips R., Swaffer B., Monis P. (2015). Integrated *Cryptosporidium* assay to determine oocyst density, infectivity, and genotype for risk assessment of source and reuse water. Appl. Environ. Microbiol..

[41] Santos S.F.O., Silva H.D., Wosnjuk L.A.C., Anunciação C.E., Silveira-Lacerda E.P., Peralta R.H.S., Cunha F.S., Ferreira T.D.S., García-Zapata M.T.A. (2016). Occurrence and evaluation of methodologies to detect *Cryptosporidium* spp. in treated water in the Central-West Region of Brazil. Expo. Heal..

[42] Maikai B.V., Baba-Onoja E.B.T., Elisha I.A. (2013). Contamination of raw vegetables with *Cryptosporidium* oocysts in markets within Zaria Metropolis, Kaduna State, Nigeria. Food Control.

[43] Rahman J., Islam Talukder A., Hossain F., Mahomud S., Atikul Islam M., Shamsuzzoha S. (2014). Detection of *Cryptosporidium* oocysts in commonly consumed fresh salad vegetables. Am. J. Microbiol. Res..

